# Rheumatoid Arthritis-Associated Autoimmunity Due to *Aggregatibacter actinomycetemcomitans* and Its Resolution With Antibiotic Therapy

**DOI:** 10.3389/fimmu.2018.02352

**Published:** 2018-10-16

**Authors:** Amarshi Mukherjee, Vanessa Jantsch, Rida Khan, Wolfgang Hartung, René Fischer, Jonathan Jantsch, Boris Ehrenstein, Maximilian F. Konig, Felipe Andrade

**Affiliations:** ^1^Division of Rheumatology, The Johns Hopkins University School of Medicine, Baltimore, MD, United States; ^2^Klinik und Poliklinik für Rheumatologie, Klinische Immunologie, Asklepios Klinikum Bad Abbach, Bad Abbach, Germany; ^3^Department of Otorhinolaryngology, University Hospital Regensburg, Regensburg, Germany; ^4^Institute of Clinical Microbiology and Hygiene, University Hospital Regensburg and University of Regensburg, Regensburg, Germany; ^5^Department of Medicine, Massachusetts General Hospital, Boston, MA, United States

**Keywords:** rheumatoid arthritis, ACPA, anti-CCP, *Aggregatibacter actinomycetemcomitans*, autoantibodies

## Abstract

**Background:** *Aggregatibacter actinomycetemcomitans* (*Aa*) is a Gram-negative coccobacillus recognized as a pathogen in periodontitis and infective endocarditis. By producing a toxin (leukotoxin A, LtxA) that triggers global hypercitrullination in neutrophils, *Aa* has been recently linked to rheumatoid arthritis (RA) pathogenesis. Although mechanistic and clinical association studies implicate *Aa* infection in the initiation of autoimmunity in RA, direct evidence in humans is lacking.

**Case:**We describe a 59-year-old man with anti-citrullinated protein antibody (ACPA)-positive RA who presented for evaluation of refractory disease. He was found to have *Aa* endocarditis. Following antibiotic treatment, joint symptoms resolved and ACPAs normalized. Given the implications for RA immunopathogenesis, we further investigated the bacterial, genetic and immune factors that may have contributed to the patient's clinical and autoimmune phenotypes.

**Methods:**DNA was extracted from serum and used to amplify the *Aa* leukotoxin (*ltx)* promoter region by PCR, which was further analyzed by Sanger sequencing. High-resolution identification of HLA alleles was performed by sequenced based typing (SBT). TNF-α, IFN-γ, GM-CSF, IL-1β, IL-6, IL-8, IL-17A, IL-18, IL-21, and IL-22 were quantified in serum by a multiplex immunoassay. IgG and IgA antibodies to *Aa* LtxA were assayed by ELISA.

**Results:***Aa* genotyping confirmed infection with a highly leukotoxic strain carrying a 530-bp *ltx* promoter deletion, shown to result in 10- to 20-fold higher bacterial expression of LtxA. Immuno-phenotyping showed high anti-LtxA antibodies, elevated cytokines implicated in RA pathogenesis (Th1/Th17), and specific host susceptibility conferred by three HLA alleles strongly linked to ACPAs and RA (DRB1^*^04:04, DRB1^*^15:01, and DPB1^*^04:01). One year after eradication of *Aa*, the patient remained free of arthritis and anti-CCP antibodies.

**Conclusion:** In the context of genetic risk for RA, systemic subacute infection with a leukotoxic strain of *Aa* can drive ACPA production and a clinical phenotype similar to RA.

## Introduction

*Aggregatibacter actinomycetemcomitans (Aa*) is a Gram-negative coccobacillus first described in 1912 as a co-isolate from actinomycosis lesions (“*Bacterium actinomycetem comitans”*) (1). *Aa* has since been recognized as a pathogen in periodontitis and, as part of the *HACEK* group, in rare cases of infective endocarditis (IE) (2–4). Recently, *Aa* has been proposed as a link between periodontitis and autoimmunity in rheumatoid arthritis (RA) due to its ability to induce citrullinated autoantigens targeted by anti-citrullinated protein antibodies (ACPAs) (5).

Leukotoxin A (LtxA) is an acylated protein toxin secreted by *Aa* and a major virulence factor in periodontitis (4). By acting as a pore-forming toxin, LtxA induces membranolysis and cell death in host immune cells, thus permitting escape from immune surveillance (4). This pathway has been shown to drive hypercitrullination of RA autoantigens in human neutrophils, thus linking *Aa* leukotoxicity to RA immunopathogenesis (5). Leukotoxic strains of *Aa* (as measured by antibodies to LtxA) are highly prevalent in RA. Exposure to *Aa* is strongly associated with ACPAs and rheumatoid factor (RF) in individuals carrying HLA-DRB1 shared epitope (SE) alleles, which confer genetic susceptibility to RA. Together, these findings have implicated *Aa* as a candidate trigger of autoimmunity in individuals at risk for RA (5). However, experimental evidence to demonstrate a causative effect is missing. Here, we report a case of early RA associated with *Aa* endocarditis and its resolution with antibiotic therapy. We believe that this case provides direct evidence that in the setting of genetic susceptibility, *Aa* is an etiologic agent that can induce ACPA production and arthritis in humans.

## Case report

A 59-year-old Caucasian man with a history of severe mitral regurgitation and recent diagnosis of seropositive RA was admitted to the hospital for evaluation of refractory joint pain and swelling. Four years prior to admission, the patient had undergone prosthetic mitral valve replacement. Since then, he had received deep dental cleanings twice a year. The patient was in his usual health until 11 months prior to admission, when he developed intermittent pain and swelling of his knees, right hip, right elbow, and wrists bilaterally that was associated with morning stiffness of >1 h. He endorsed 11 lbs. weight loss and night sweats, but no fevers. Following 6 months of persistent symptoms, the patient saw a local rheumatologist who noted synovitis of the 2nd left metacarpophalangeal joint and tenosynovitis of the extensor tendons of his left hand. Laboratory workup showed evidence of systemic inflammation [C-reactive protein (CRP) 100 mg/L, erythrocyte sedimentation rate (ESR) 84 mm/h] and positive ACPAs (measured by the anti-CCP antibody assay). Testing for RF was negative. The patient was diagnosed with early seropositive RA, and he was started on immunosuppression with prednisolone and methotrexate. Given lack of clinical improvement, leflunomide was added. Due to persistent joint pain and swelling, the patient was hospitalized 2 months later for evaluation.

**Figure 1:**
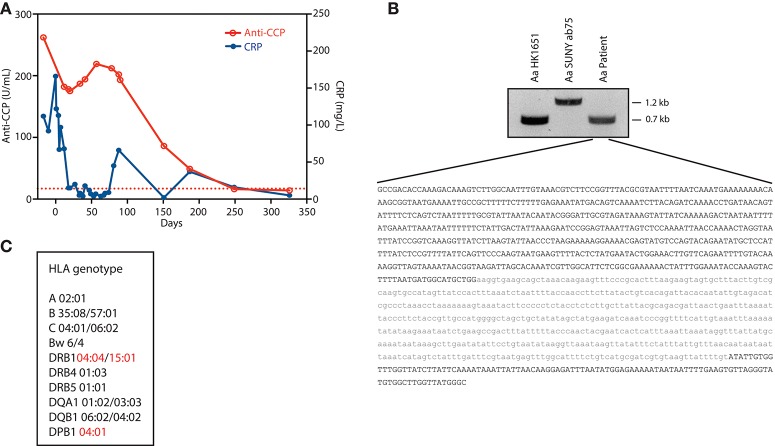
Autoimmune, genetic, and microbial factors associated with *Aa*-induced early RA. **(A)** Serial measurements of C-reactive protein (CRP) and anti-cyclic citrullinated peptide (CCP) antibodies. Day 0 marks the diagnosis of *Aa* endocarditis. Anti-CCP was determined in serum using an automated electrochemiluminescence immunoassay platform (Elecsys, Roche). The dotted line marks the cut-off for anti-CCP antibody positivity (17 U/mL). **(B)** DNA extracted from patient serum was used to amplify the *Aa ltx* promoter region by PCR. PCR products of strains *Aa* HK1651 and *Aa* SUNY ab75 were used as a reference for JP2 and non-JP2 clones, respectively. The PCR product amplified from patient serum was analyzed by Sanger sequencing. The resulting nucleotide sequence is shown in black. The missing nucleotide sequence corresponding to the 530-bp deletion (Δ530) previously described in *Aa* JP2 clones is shown in gray. **(C)** HLA genotype. HLA risk alleles linked to ACPA production and RA are marked in red.

At the time of hospital admission, laboratory evaluation showed CRP 112 mg/L, ESR 79 mm/h, and high-titer anti-CCP IgG antibodies (262 U/mL; reference range <17 U/mL). Musculoskeletal ultrasound (US) showed effusion of the 2nd and 3rd right proximal interphalangeal joints as well as 1st and 4th right metatarsophalangeal joints. There was evidence of tenosynovitis of the right wrist extensor tendons, and inflammation of the flexor tendons of the right ankle and right Achilles tendon. Radiographs of the hands and feet showed no erosions. Prednisolone was increased. The patient was started on etanercept, and leflunomide was discontinued. Following a brief period of improvement, the pain around the right Achilles tendon and right wrist flexor tendons worsened within 3 weeks. US revealed new abscess formation along the right Achilles tendon. Incision and drainage was performed with wound cultures demonstrating *Aa* by PCR and sequence analysis. Blood cultures grew *Aa* in 2/3 sets of bottles, and echocardiography confirmed prosthetic mitral valve endocarditis. All immunosuppressive medications were discontinued, and antibiotic therapy with ceftriaxone 2 g IV daily was started. CRP levels decreased, and the joint pain improved. After completing a 6 week course of intravenous antibiotics, the patient's joint pain and swelling had resolved. Thereafter, anti-CCP antibody levels started to decline (Figure 1A). A non-ulcerated squamous cell carcinoma of the tongue was subsequently diagnosed and treated with radiotherapy. At ~1 year follow-up, the patient remained free of joint symptoms, and anti-CCP antibodies and CRP levels had normalized (anti-CCP 13.5 U/mL; CRP 5 mg/L) (Figure 1A).

## Materials and methods

### *Aa* ltx promoter genotyping

DNA extracted from patient serum (QIAamp DNA kit, Qiagen), and DNA from strains *Aa* HK1651 (serotype b, ATCC 700685) and *Aa* SUNY ab75 (serotype a, ATCC 43717) were used to amplify the *Aa ltx* promoter region by PCR using primers *ltx3*/*ltx4* (*ltx3*: *5*′*-GCCGACACCAAAGACAAAGTCT-3*′ and *ltx4*: *5*′*-GCCCATAACCAAGCCACATAC-3*′) as previously described (6). *Aa* strains HK1651 and SUNY ab75 were used as a reference for JP2 and non-JP2 clones, respectively. The PCR product amplified from patient serum was analyzed by Sanger sequencing in both 5′ and 3′ directions using the *ltx3* and *ltx4* primers, respectively.

### HLA typing

Sequenced based typing (SBT) was used for high-resolution identification of alleles of HLA-A, -B, -C, - DRB1, -DQB1, and -DPB1. SBT uses PCR to amplify the locus of interest and Sanger sequencing to determine the nucleotide sequence. HLA-typing was performed at the Johns Hopkins University Immunogenetics Laboratory.

### Quantification of cytokines and Anti-LtxA antibodies in serum

Serial measurements of TNF-α, IFN-γ, GM-CSF, IL-1β, IL-6, IL-8, IL-17A, IL-18, IL-21, and IL-22 were quantified in patient serum by multiplex immunoassay (Meso Scale Diagnostics). IgG and IgA antibodies to LtxA were assayed in serial serum samples by ELISA as previously described (5) using a recombinant immunodominant peptide of LtxA (amino acids 730-1055) as antigen (5).

## Results

### Bacterial virulence of *Aa*

The virulence factor LtxA generates citrullinated autoantigens in human neutrophils (5). Expression of LtxA varies considerably among *Aa* strains and is regulated by both environmental factors and genetic variation within the *ltx* promoter (7, 8). To define potential variations in the *ltx* promoter region associated with highly virulent strains, bacterial DNA from patient serum was amplified by PCR (Figure 1B). The amplified *ltx* promoter contained a 530-bp deletion (Δ530), which has been shown to result in 10- to 20-fold higher expression of LtxA compared to wild-type *Aa* strains due to deletion of a transcriptional terminator (6, 8, 9). These data indicate that the patient was infected with a highly leukotoxic strain of *Aa*.

### Genetic susceptibility

Since the presence of autoantibodies in RA patients carrying HLA-DRB1 susceptibility alleles is strongly linked to *Aa* exposure (5), HLA-typing was performed at high resolution (Figure 1C). The patient's haplotypes revealed at least 3 HLA alleles strongly associated with seropositive RA: two heterozygous DRB1 risk alleles (^*^04:04 and ^*^15:01) and one homozygous DPB1 risk allele (^*^04:01) (10, 11). DRB1^*^04:04 confers strong disease susceptibility by the presence of residues Val, Arg and Ala at positions 11, 71, and 74, respectively. In DRB1^*^15:01 and DPB1^*^04:01, risk is defined by amino acid positions 74 (Ala) and 9 (Phe), respectively. These residues are located within the antigen-binding groove of the HLA class II molecule (10), and may facilitate presentation of citrullinated self-peptides (12).

### Inflammatory milieu

**Figure 2:**
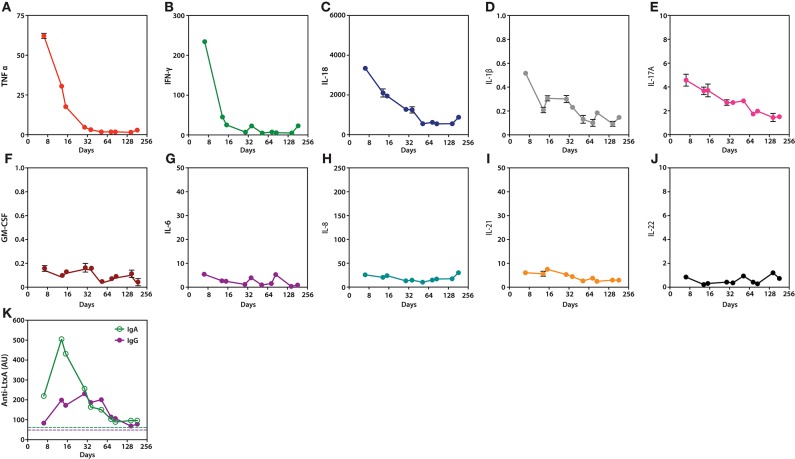
Serum cytokines and anti-LtxA antibodies during *Aa* infection and after initiation of antibiotic treatment. **(A–J)**, Serial measurements of TNF-α, IFN-γ, IL-18, IL-1β, IL-17A, GM-CSF, IL-6, IL-8, IL-21, and IL-22 (pg/mL), respectively. **(K)** Serial quantification of IgG and IgA antibodies to LtxA. Dotted lines mark cut-offs for anti-LtxA positivity, which were established using a cohort of healthy individuals without periodontitis (5). Cytokine and anti-LtxA antibody levels were assayed in triplicate and duplicate, respectively. Mean values are shown. Day 0 marks the diagnosis of *Aa* endocarditis.

To define the inflammatory milieu that may have contributed to loss of immune tolerance and arthritis, a panel of cytokines was quantified in samples collected longitudinally after diagnosis of *Aa* infection (Figures 2A–J). The cytokine profile induced by *Aa* was characterized by expression of several cytokines implicated in autoimmunity and RA (13). These included TNF-α, IFN-γ, IL-1β, IL-17A, and IL-18 (Figures 2A–E). Levels decreased progressively within weeks to months after initiation of antibiotic treatment, which coincided with clinical improvement. Other cytokines did not show similar dynamics, including GM-CSF, IL-6, IL-8, IL-21, and IL-22 (Figures 2F–J).

### Humoral immunity to *Aa*

Although IgA and IgG antibodies to LtxA were positive at the time of IE diagnosis, levels markedly increased several days after initiation of antibiotic treatment (Figure 2K). This coincided with a decline in peripheral cytokine levels (Figures 2A–E). The increase in anti-LtxA antibody levels suggests a more efficient humoral immune response against *Aa*, which may have aided pathogen clearance. Anti-LtxA antibody levels decreased with time, but remained positive at lower titers. Similar dynamics of the anti-*Aa* antibody response have previously been reported with initiation of periodontal treatment (14).

## Discussion

Autoimmune diseases result from the interplay of genetic susceptibility, environmental factors, and stochastic events that together determine an individual's risk of developing disease. Loss of tolerance to peptidylcitrulline is an immune hallmark of RA (15), and pathogens with the potential to provoke autoantigen citrullination have emerged as putative agents that may trigger or sustain the anti-CCP response in RA (16). An association between RA and *Aa* is supported by mechanistic and clinical evidence (5). We believe this case provides proof of concept that in the genetically predisposed individual, *Aa* can induce the autoimmune responses associated with RA. The resolution of RA-associated symptoms (i.e., morning stiffness, polyarthritis, tenosynovitis) and anti-CCP antibodies with antibiotic therapy further supports an etiological role of *Aa* in driving autoimmunity in this patient.

*Aa* endocarditis is an insidious disease with a subacute or chronic course, which can have a prolonged period of symptoms before diagnosis (up to 60 weeks) (17). The presence of prosthetic heart valves, oral infection and dental procedures are among the risk factors associated with this illness (17). Since periodontal infection was not documented in the case presented here, it is possible to suggest that *Aa* may have reached the vascular compartment as result of inoculation during deep dental cleaning. Different to other cases of *Aa* endocarditis that present with prominent systemic features (fever, weight loss, rash, hepatosplenomegaly, hematological abnormalities, and hematuria) and often severe complications such as heart failure, renal failure, mycotic aneurysms, and septic embolization (17), the clinical course in this patient was exceptionally defined by arthritis and anti-CCP antibodies.

It is possible that several factors may have contributed to the patient's risk of developing loss of tolerance to peptidylcitrulline and RA-associated symptoms in the setting of (sub-)acute *Aa* infection. First, we identified a strong genetic susceptibility for seropositive RA as conferred by 3 distinct HLA-class II risk alleles in 2 loci (DRB1^*^04:04, DRB1^*^15:01, and DPB1^*^04:01). Distinct alleles are thought to confer additional disease risk by expanding the repertoire of possible RA autoantigens presented on HLA-class II molecules (18). In this patient, the different risk alleles likely conferred independent susceptibility to RA through the presence of distinct amino acid residues within the HLA class II binding groove (10).

Second, the patient was found to be infected with a highly leukotoxic *Aa* strain that carries a Δ530 *ltx* promoter deletion (8). This genotype of *Aa*, first described in the highly leukotoxic strain JP2 (8), is endemic in various populations originating in North and Central Africa and associated with aggressive periodontitis (4). The prevalence of JP2-like strains in *Aa* endocarditis is unknown, but to our knowledge, no cases are reported to date. In a study of 35 blood isolates of *Aa*, no JP2 promoter deletions were identified (19). As the generation of citrullinated autoantigens by *Aa* is dependent on LtxA alone (5), the identification of the highly leukotoxic JP2 genotype may explain why loss of immunologic tolerance to citrullinated proteins and clinical autoimmunity occurred in this patient. Although certain autoantibodies including RF are common in IE, anti-CCP antibodies have only been reported in one case of IE in which the pathogen was not identified (20). Indeed, the unlikely co-incidence of infection with a rare, highly virulent genotype of *Aa* in a patient uniquely predisposed to make ACPAs may explain why anti-CCPs and RA have not been reported in other cases of *Aa* endocarditis.

A third factor that may have facilitated the induction of arthritis is the unique pro-inflammatory milieu associated with *Aa* JP2 infection. *Aa* serotype b, which includes JP2 clones (8), is a potent inducer of Th1 (TNF-α and IFN-γ) and Th17 (IL-17A) immune phenotypes (21). LtxA also induces production of IL-1β and IL-18 by macrophages (22). These cytokines, which are central mediators in the immunopathogenesis of RA (13), also characterized the systemic cytokine profile identified in this patient during active *Aa* infection (Figures 2A–E). Although it is difficult to define whether these cytokines played a role in the patient's clinical presentation, it is interesting that although the addition of etanercept transiently improved the joint symptoms, it may also have allowed for abscess formation which help to unmask the underlying infection with *Aa*. Thus, it is possible that the cytokine milieu induced by the *Aa* JP2 strain helped to limit bacterial dissemination and the development of common complications associated with *Aa* endocarditis, but also contributed (together with *Aa*-induced anti-CCPs) to the development of arthritis in this patient. TNFα blockade may indeed have changed the clinical course of the disease from an indolent infection with RA phenotype to a more typical presentation of *Aa* endocarditis (e.g., septic emboli).

Although the patient fulfilled the 2010 ACR/EULAR classification criteria for RA [8/10 points based on synovitis of at least 4 small joints (3 pt.); ACPA >3xULN (3 pt.); duration >6 weeks [1 pt.]; and abnormal CRP and ESR (1 pt.)] (23), his disease course is not classic. In RA, the transition from pre-clinical autoimmunity to overt arthritis often takes several years (24). This is consistent with a model of indolent infection such as periodontitis or bronchiectasis leading to chronic antigenic simulation (25). In cases of RA potentially associated with *Aa* periodontitis, the production of citrullinated autoantigens would represent a chronic, localized process which may result in long preclinical phase. During this phase, amplification pathways may be established that maintain disease. In this case of systemic *Aa* infection, widespread production of RA autoantigens triggered by a highly leukotoxic strain of *Aa* may have resulted in acute autoantibody production and arthritis, which rapidly resolved after the driver of autoantigen production (i.e., *Aa*) was eradicated.

It is now appreciated that the relationship between infectious agents and autoimmune arthritis is more complex than the one pathogen-one disease model that built the conceptual framework for Koch's postulates. The existence of a single pathogen that acts as a driver of autoimmunity in all patients with RA is unlikely. Similarly, only a fraction of individuals infected with a microbial species of arthritogenic potential will develop RA. This case underscores the importance of both genetic susceptibility and environmental agents for the induction of autoimmune pathology, and in a human model provides direct evidence that leukotoxic strains of *Aa* can trigger autoimmune features found in RA. Moreover, it suggests that in some cases, this pathogen can be a reversible cause of RA. Although the idea of definitive treatment of early autoimmunity in RA is enticing, it remains uncertain whether established RA associated with *Aa* periodontitis may be modifiable by antibiotic therapy.

## Ethics statement

The patient gave written informed consent prior presentation of the case. The case report is not part of a clinical trial.

## Author contributions

AM, JJ, and RK performed *in vitro* studies. VJ, WH, RF, and BE clinically followed the patient. FA supervised experiments. MK and FA wrote the manuscript and prepared the figures. All authors contributed to the preparation of the final manuscript.

### Conflict of interest statement

FA received a grant from Medimmune and is author on issued Patent No. 8,975,033 entitled “Human Autoantibodies Specific for PAD3 which are Cross-reactive with PAD4 and their Use in the Diagnosis and Treatment of Rheumatoid Arthritis and Related Diseases.” FA has served as consultant for Bristol-Myers Squibb Company and Pfizer. The remaining authors declare that the research was conducted in the absence of any commercial or financial relationships that could be construed as a potential conflict of interest. The reviewer YP and handling Editor declared their shared affiliation at the time of the review.
